# Mapping breast and prostate cancer in the Brazilian public health system: study protocol of the Onco-Genomas Brasil

**DOI:** 10.3389/fonc.2024.1350162

**Published:** 2024-03-13

**Authors:** Jaqueline Bohrer Schuch, Cláudia Bordignon, Mahira Lopes Rosa, Angélica Cerveira de Baumont, Marina Bessel, Gabriel S. Macedo, Daniela Dornelles Rosa

**Affiliations:** ^1^ Responsabilidade Social, Hospital Moinhos de Vento, Porto Alegre, RS, Brazil; ^2^ Molecular Pathology, Rede D'Or São Luiz, São Paulo, Brazil; ^3^ D'Or Institute for Research and Teaching (IDOR), São Paulo, Brazil

**Keywords:** exome sequencing, breast neoplasms, prostatic neoplasms, cancer, next-generation sequencing

## Abstract

**Background:**

Breast and prostate cancers are the most common malignancies diagnosed in women and men respectively, and present with great clinical heterogeneity, even in tumors with the same histology and same site of origin. Somatic and germline molecular alterations in DNA may have prognostic and predictive impact, influencing response to therapies and overall survival. Our aim is to characterize the somatic and germline genomic landscape of women with locally advanced HER2-positive breast cancer and men with metastatic prostate cancer in Brazil. Secondarily, we aim to identify genetic variants associated with tumor prognosis and treatment response, identify patients carrying pathogenic alterations in cancer-predisposing genes, and characterize the genetic ancestry of the population included in the study.

**Methods:**

This observational multicenter cohort study will include 550 adult patients from the five macro-regions of Brazil, divided into two arms: 1) breast cancer and 2) prostate cancer. Clinical and pathological data will be collected, as well as DNA samples from peripheral blood and tumor samples. In arm 1, the inclusion criteria are a histological diagnosis of breast carcinoma with overexpression of HER-2, clinical stage II or III, and current neoadjuvant treatment with chemotherapy plus trastuzumab. In arm 2, the criterion is a histological diagnosis of prostate adenocarcinoma, clinical stage IV. Whole-exome sequencing (WES) will be performed to identify variants that may be drivers and/or actionable in a specific patient or tumor. These variants will be interpreted and classified according to their population frequencies, *in silico* predictors, functional studies, and literature data, following international guidelines proposed by expert societies.

**Discussion:**

This trial will contribute to the construction of a robust database that should provide a better understanding of the genomic profile of patients with breast and prostate cancer in Brazil. Considering the miscegenation of the Brazilian population, knowledge generated from these data will have implications for future studies of this specific population.

**Clinical trial registration:**

[clinicaltrial.gov], identifier [NCT05306600]

## 1 Introduction

Cancer is a global public health problem with major economic and health implications. Breast and prostate cancers are the most common neoplasms diagnosed in women and men respectively (excluding non melanoma skin cancer). Recent worldwide estimates indicate more than 2,260,000 and 1,410,000 new cases per year of breast and prostate cancers, respectively. In Brazil, the incidence of each of these two types of cancer exceeds 70,000 cases per year (1, 2).

Approximately 20% of breast cancers overexpress the HER2 receptor (HER2-positive tumors) (3, 4), which is associated with an aggressive phenotype and worse survival outcomes (5). This scenario is similar in Brazil (4). Standard systemic treatment for locally advanced disease consists of chemotherapy and 12 months of anti-HER2 therapy, resulting in a 30% reduction in mortality (6). Neoadjuvant treatment is the preferred approach for stages II and III HER2-positive breast cancers, to increase the probability of pathologic complete response (pCR) which is associated with better outcomes (7–9). Current treatment guidelines indicate the use of pertuzumab during neoadjuvant treatment, and trastuzumab emtansine during adjuvant treatment. However, in the public health system in Brazil, access to this treatment protocol is limited. Therefore, the identification of genetic alterations that increase the risk of insufficient tumor response to the available neoadjuvant anti-HER2 treatment (trastuzumab) in the Brazilian population can help direct resources to improve treatment in patients who need it most.

Regarding prostate cancer, approximately 10 to 20% of individuals are diagnosed with metastatic disease or develop metastases after treating the primary tumor. Progression to metastatic disease is incurable and significantly impacts quality of life, especially due to bone metastases, which are present in approximately 90% of patients with stage IV prostate cancer (10, 11).

Malignant solid tumors exhibit great clinical heterogeneity, and even those with the same histology and site of origin may respond differently to treatment. Somatic and germline molecular alterations in DNA have prognostic and predictive impact, influencing response to therapies and overall survival. For instance, detection of mutations in the *BRCA1* and *BRCA2* genes, besides allowing appropriate genetic counseling and individualized management of patients diagnosed with cancer and their families (12), can be associated with a more aggressive presentation and worse outcomes in prostate cancer (13) and response to targeted therapies in prostate and breast neoplasms (14, 15).

New technologies for genomic sequencing have helped to elucidate somatic molecular alterations present in different cancer types and can be used to guide treatment with targeted drugs. In addition, germline DNA sequencing may be applied to identify individuals at high risk of cancer and, subsequently, support clinical management and strategies for the prevention and early detection of new cancer cases. Whole-exome sequencing (WES) involves the study of the coding regions (exons) of DNA, which represent about 1.5% to 2% of the human genome. WES is a complex test that sequences and analyzes around 180,000 exons in nearly 22,000 genes and aims to identify variants (germline or somatic) that may be drivers and/or actionable in a specific patient or tumor. In view of the high prevalence of breast and prostate cancers, the molecular characterization of these tumors – and its relationship with response to routine treatment – may allow the identification of patients with a better prognosis.

The primary aim of this study is to characterize the somatic and germline genomic landscape of women with locally advanced HER2-positive breast cancer and men with metastatic prostate cancer in Brazil. We also aim to identify genetic variants associated with tumor prognosis and treatment responses, as well as to identify patients carrying pathogenic alterations in cancer-predisposing genes. Finally, the study also aims to characterize the genetic ancestry of the analyzed population.

## 2 Methods

### 2.1 Design

Observational multicenter cohort study including women with breast cancer and men with prostate cancer, recruited from all five macro-regions of Brazil, so as to maximize representativeness of the Brazilian population. This study will collect clinical, pathological, and biological data from 550 patients assisted by the Brazilian public Unified Health System. Peripheral blood samples and tumor samples from formalin-fixed paraffin-embedded (FFPE) specimens will be collected for WES analyses. This will be a two-arm study: arm 1 will include 300 patients with HER2-positive breast cancer and arm 2 will include 250 patients with metastatic prostate cancer. The period for patient recruitment occurs between October 2022 and the end of December 2023.

### 2.2 Recruitment and sample size

Institutions that care for patients in the public Unified Health System and that have High-Complexity Oncology Care Centers (*Centros de Alta Complexidade em Oncologia*, CACONS) or High-Complexity Oncology Units (*Unidades de Alta Complexidade em Oncologia*, UNACONS) will be pre-selected by the research team of the coordinating center. Medical oncologists and medical researchers from these institutions will be contacted and will complete a feasibility assessment questionnaire to confirm their interest and the necessary requirements for carrying out the study. We expect to include 20 to 30 institutions (at least one from each of Brazil’s five geopolitical regions). Institutions that meet the following criteria will be eligible:

- Care for patients through the Unified Health System;
- Demonstrate high recruitment potential;
- Have a research team available for training in the study protocol and in Good Clinical Practice (GCP);
- Have a High-Complexity Oncology Care Center (CACONS) or a High-Complexity Oncology Unit (UNACONS);
- Accept participation in the study by signing a cooperation agreement;
- Have a medical oncologist available to be a principal investigator (PI) at the participating center.

The study sample will consist of 550 patients: 300 in arm 1 (breast cancer) and 250 in arm 2 (prostate cancer). The sample size will be divided according to projections by the Brazilian Institute of Geography and Statistics (IBGE) for the distribution of the Brazilian population within the five macro-regions, considering data from July 2021. The selection of participating centers will be distributed to ensure proportionality with the population density of each region. The total sample size of the study was defined using the information that approximately 20% of breast cancers overexpress the HER2 receptor, with an acceptable difference of 5%, a design effect of 2, a confidence level of 95%, and 80% statistical power. The inclusion of participants in each center will take place by convenience, considering a consecutive and prospective sample.

The inclusion criteria for participants in Arm 1 (breast cancer) are:

- Women aged ≥ 18 years;
- Histological diagnosis of breast carcinoma with overexpression of HER-2 (HER-2 with 3+ or 2+ on immunohistochemistry and presence of positive *in-situ* hybridization [ISH]);
- Clinical stage II or III according to the American Joint Committee on Cancer (AJCC) classification;
- Current neoadjuvant treatment with chemotherapy plus trastuzumab – accepted chemotherapy regimens: anthracycline (doxorubicin or epirubicin) followed by taxane (docetaxel or paclitaxel), combined with trastuzumab, or an anthracycline-free regimen composed of taxane (docetaxel or paclitaxel) plus carboplatin and trastuzumab;
- Written informed consent.

The inclusion criteria for participants in Arm 2 (prostate cancer) are:

- Men aged ≥ 18 years;
- Histological diagnosis of prostate adenocarcinoma;
- Clinical stage IV according to the AJCC classification;
- Written informed consent.

Exclusion criteria for both arms consist of the absence of tumor tissue from FFPE and the impossibility of collecting peripheral blood for genomic evaluation. For the breast arm, the FFPE must refer to the diagnostic biopsy, prior to neoadjuvant treatment and surgery. For the prostate arm, the FFPE must not be from bone tissue, only from soft tissue (prostate or other tissues affected by metastases).

### 2.3 Measurements

The research protocol includes a questionnaire with demographic data, personal, and family cancer history. In arm 1 (breast cancer), a specific questionnaire about the neoplasm, consisting of data from the initial biopsy, presence of estrogen and progesterone receptors, HER2 positivity, menopausal status, tumor location, and staging, will be administered. Data on neoadjuvant treatment, including the HER2 blockade and the chemotherapy regimen, will also be collected. A second questionnaire to collect data on surgery, treatment response, and adjuvant therapy will then be applied. A third questionnaire with participant follow-up data will be completed at the end of study enrollment, with updated information on relapse, further treatment, and survival.

In arm 2 (prostate cancer), a questionnaire with data on tumor characteristics will also be applied. This questionnaire will include items on the initial biopsy, staging, date of diagnosis of metastatic disease, treatments performed (prostatectomy, radiotherapy, hormonal blockade), and use of additional therapies. As in arm 1, a second questionnaire with participant follow-up data will be completed at the end of study enrollment, with updated information on further treatment and vital status.

### 2.4 Outcomes

The characterization of mutations in somatic and germline landscape of women with locally advanced HER-2 positive breast cancer and men with metastatic prostate cancer is the primary study outcome. The secondary outcomes consist of a description of the response to treatment and associated phenotypic data, identification of the prognostic and predictive impact of molecular alterations, presence of germline mutations in genes predisposing to cancer, and the measure of the ancestry of individuals included in the study.

### 2.5 Procedures

Participants will be selected on the basis of a screening process carried out at participating centers. Those who meet the eligibility criteria will be invited to participate in the study by a central investigator. The objective is to achieve a population-based cohort of women with locally advanced HER2-positive breast cancer, with an indication for neoadjuvant treatment with targeted anti-HER2 therapy, and of men with metastatic prostate cancer.

After signing the informed consent form, sociodemographic and clinical questionnaires including information about cancer treatment history and response will be collected through an interview during the participant’s visit as well as from medical records. The participant will be referred for the acquisition of biological samples. A blood sample from eligible participants will be collected at enrollment. FFPE tumor specimens, as well as the respective histological slides, will be obtained from previous biopsies performed for clinical purposes. No new samples will be performed to obtain tumor tissue.

At the end of study enrollment, follow-up data will be collected, including information on survival, oncologic therapy, and response to treatment. These data will be extracted from medical records and do not require further consultation of the participant. All clinical data will be entered into the REDCap platform.

### 2.6 Biological processing

All biological materials collected for this study will be sent from the participating centers to the coordinating center for processing and storage. Transport will be carried out by a specialized company. The material will be placed in plastic packages within a Styrofoam^®^ box containing reusable ice packs. The biological material received will be registered in the internal system of the Pathology laboratory at Hospital Moinhos de Vento (HMV), where the samples will be processed and stored. After sample processing at HMV, the germline and tumor DNA samples will be sent to the Clinical laboratory at Hospital Israelita Albert Einstein (HIAE).

The processing of biological material is described below in greater detail.

#### 2.6.1 Tumor samples - collection, diagnosis confirmation, and DNA extraction

Tumor samples will be obtained from FFPE material considering the previously mentioned specifications. Histologic slides from paraffin blocks should preferably be sent along with the FFPE material. The original anatomic pathology report must be sent for review and diagnostic confirmation. These materials should be organized by a researcher at the participating center and sent to the coordinating center.

The original identification present on the FFPE material and histological slides should not be erased. These data will be registered in the REDCap platform and in the Pathology laboratory system since this material will be returned to the participating centers at the end of the study.

The FFPE material and histological slides will be examined to confirm the diagnosis (breast cancer with overexpression of HER-2 or prostate adenocarcinoma). If necessary, new anatomopathological and immunohistochemical slides can be made for this purpose. The reviewed reports will be sent to the operational team of the coordinating center. If the diagnosis is not confirmed, the team will contact the principal investigator of the participating center to notify of the participant’s ineligibility. If the diagnosis is confirmed, the material will be processed at the HMV Molecular Biology Laboratory. For the DNA extraction, ten 10-µm sections will be prepared. Tumor regions will be identified and marked by a pathologist and subjected to needle microdissection under a stereomicroscope to ensure a tumor cell content >20%. Genomic DNA from the tumor will be extracted with a commercial kit (QIAamp DNA FFPE Tissue, Qiagen). A DNA fragmentation analysis will also be performed to verify the quality of the material obtained on the Tapestation equipment. The minimum quality parameters of FFPE DNA for WES are medium fragment sizes of 800bp, peak of fragmentation of the least 350bp, and the minimum amount of material ranging from 250 ng to 500 ng in total.

Microtubes containing DNA will be identified with the REDCap code followed by gender, year of birth, and the letter S (indicating the source of the material, i.e., somatic). These samples will be stored at -20°C until sequencing. The remaining FFPE material as well as the histologic slides will be stored in the Pathology laboratory storage room and will be returned to the participating centers at the end of the study.

#### 2.6.2 Blood samples - collection and DNA extraction

Peripheral blood will be collected into 8.5 mL PAXgene tubes by the local team at each participating center. The tubes will be labeled with the code generated by REDCap for each participant, followed by gender and year of birth. Samples will be sent as soon as possible after collection (preferably immediately) and kept refrigerated until shipping. Blood samples will be received at the HMV laboratory and sent to the Molecular Biology sector for DNA extraction using QIAamp DNA Blood kits (Qiagen). The minimum quality parameters for whole-blood DNA are a 260:280 absorbance ratio between 1.8-2.0 (NanoDrop) and a minimum volume of 20 µL with a minimum concentration of 35 ng/µL, quantified by Qubit. As for DNA from tumor samples, microtubes containing DNA will be identified with the REDCap code followed by gender, year of birth, and the letter G (indicating the source of the material, i.e., germline). These samples will be stored at -20°C until sequencing.

#### 2.6.3 Whole-exome sequencing

DNA samples from the participants’ tumor and blood specimens will be sent to the Clinical laboratory at HIAE for WES analysis. Samples will be transported (in Styrofoam^®^ coolers refrigerated at 4°C) by a specialized contractor. The coordinating center team will register the samples in the HIAE Matrix system before shipment, generating a manifest with the list of registered samples. This manifest will be printed and sent with the samples.

In addition, part of these DNA samples (somatic and germline) will remain stored at the HMV laboratory and later sent to the Brazilian Ministry of Health for incorporation into the National Program of Genomics and Precision Health (*Genomas Brasil*).

For germline and tumor DNA samples, the following quality parameters will be applied: percent contamination up to 0.02 and minimum Q30 of 85%. Furthermore, the average size of the DNA library obtained by Tapestation should be between 375 and 450 base pairs. In tumor DNA samples, this may be slightly smaller considering the initial fragmentation of the DNA sample. The minimum size of the sequenced target DNA fragment is estimated to be between 100 and 150 base pairs for tumor and germline samples.

An average coverage of at least 50X will be applied to germline DNA samples and at least 250X for somatic DNA samples. Next-generation sequencing (NGS) will be performed on the Illumina NovaSeq6000 platform. Process steps include the construction of libraries using the Twist Library Preparation EF 2.0 kit, the Twist Target Enrichment Standard Hybridization v2 kit for enrichment, and NOVASEQ 6000 S4 reagent kit for sequencing. The sequencing, processing, and evaluation of raw data will be performed in pairs, evaluating tumor and germline samples from the same participant in the same run. Samples that do not meet the sequencing quality criteria will be evaluated individually to identify the probable cause.

For each sequenced exome, FASTQ files will be generated from the raw sequencing data using the bcl-convert v3.9.3 program. The DRAGEN v.3.10.4 program will be used to map and aligned the genetic sequences produced by WES with the hg38 reference human genome, with marking of duplicated sequences for extraction of germline variants such as single nucleotide variants (SNV), insertion/deletions (InDel), and copy number variations (CNV). The analyzed regions will be limited to the coordinates present in the BED file of the panel used for sequencing plus 10 base pairs at both ends. The generated VCF files containing the variants will be annotated on the Varstation^®^ platform with national and international reference public databases and will be available for analysis and visualization on the platform, along with the BAM files. For evaluation of pathogenicity, conservation and splicing functional predictors will be used, including REVEL, BayesDel, phyloP, and dbscSNV. The classification of germline variants will follow the criteria established by the American College of Medical Genetics and Genomics (ACMG) and by ClinGen (Clinical Genome Resource).

For tumor DNA assessment, the pipeline will be very similar, and will include analysis of paired exomes in DRAGEN v.3.10.4. The sequences will be mapped with the reference human genome (hg38) with duplicated sequence marking for extraction of somatic variants of the SNV, InDel, and tumor mutation burden (TMB) and microsatellite instability (MSI) biomarkers. The next pipeline steps will follow the same flow as the germline DNA sample. The generated VCF files containing the variants will be annotated on the Varstation^®^ platform; variants with allele frequencies equal to or greater than 5% will be considered. The classification of somatic variants will follow the criteria established by the Association for Molecular Pathology (AMP), and actionability will be evaluated by searching the OncoKB database.

Global ancestry inference will be performed using principal components analysis (PCA) and ADMIXTURE software. From a multi-sample VCF file containing all the target samples and a BED file containing the coordinates of the sequencing kit used, only those SNVs in common between the target samples and the samples from the reference panel will be selected. Samples from the 1,000 Genomes phase 3 project will be used as a reference to determine continental African (AFR), East Asian (EAS), and European (EUR) ancestry supergroups. This analysis will generate a.tsv file containing the calculated principal components for each sample, and another.tsv file containing the genome fraction assigned to each of the three population groups for each sample. It bears stressing that inference of global ancestry is usually performed using thousands of genomic markers, and the same level of reliability cannot be guaranteed with an analysis based on exon data alone.

### 2.7 Genetic reports and counseling

WES data will be analyzed by molecular geneticists specializing in the interpretation of cancer-related genomic data. Alterations identified in the exome will be evaluated, interpreted, and classified according to their population frequencies, *in silico* predictors, functional studies, and literature data, following international guidelines proposed by expert societies. Germline variants will be classified into one of five recommended categories: benign, likely benign, variants of uncertain significance, likely pathogenic, and pathogenic. Reports containing the results of the exome analysis will be issued to the local research physicians at each participating center, who will then forward them to the participants.

In cases where mutations classified as pathogenic and/or likely pathogenic are detected, genetic counseling will be provided by the geneticist at the coordinating center to the oncologist at the participating center. Counseling will address the genetic alterations found and respective suggestions for management and prognosis. The oncologist at the participating center should inform the participant of these findings and their implications.

### 2.8 Data analyses and statistics

Categorical variables will be expressed as absolute and relative frequencies. Continuous variables will be presented as means and standard errors or medians and interquartile ranges, as appropriate for their distribution. Chi-square, Fisher’s exact, Mann–Whitney *U*, or *t*-tests will be used to test for associations between clinical and biological variables. Bonferroni correction will be applied when appropriate.

Univariate and multivariate analyses will be conducted to evaluate the relationship between outcome and predictor characteristics, such as specific genes or mutations that have an effect on the investigated outcomes, including tumor prognosis and treatment response. PCA will be used to summarize multidimensional data into fewer variables. This approach is widely used to identify and adjust for ancestry differences among individuals.

Statistical analyses will be performed using RStudio software (R Foundation for Statistical Computing, Vienna, Austria; http://www.R-project.org), considering a significance level of 5% for alpha error (p<0.05).

### 2.9 Monitoring and quality control

The quality of data collected and reported to the coordinating center will be systematically monitored. Monitoring activities will be carried out following the ALCOA principle, i.e., verifying that the data are attributable, readable, contemporary, original, and accurate in relation to the source documents. Monitoring will take place remotely, individually for each participating center, every 3 months. Centralized monitoring will identify missing data and distinguish between reliable and potentially dubious data. Furthermore, each center’s characteristics and performance metrics will be reviewed weekly to identify systematic or significant errors in data collection and reporting. Any protocol deviations identified will be evaluated to determine if action is required. Appropriate measures to prevent the recurrence of such deviations will be adopted by investigators at participating centers.

All transport of biological samples will be monitored on a daily basis to ensure proper packaging, shipping, and receipt. The sample processing flow at HMV and HIAE will also be monitored in relation to protocol compliance, quality of activities, and adherence to previously established deadlines.

All investigators involved in the study will be trained by the coordinating team and will be required to produce up-to-date GCP certification.

## 3 Discussion

Breast and prostate cancer are among the most prevalent neoplasms worldwide. The identification of molecular alterations that may identify patients at high risk of cancer, predict response to treatments, or define prognosis is important to inform strategies for the better management of patients with these malignancies. This will be the first study to identify genetic variants associated with these outcomes in any Brazilian population diagnosed with locally advanced HER2 breast cancer and metastatic prostate cancer. In addition, the identification of genetic alterations that are related to hereditary syndromes has the potential to help prevent new tumors at the individual patient level.

Nonetheless, this project has some limitations. Participants will be included consecutively and competitively among the participating centers. Although several hospitals from all Brazilian macro-regions will be contacted and invited to participate in the study, there is a risk that, in some regions, the sample size will not be reached due to limited inclusion capacity in each center. Furthermore, the study will only analyze the exome of the participants, not their entire genome; non-coding and intergenic regions will not be included in the analysis. However, DNA samples and clinical and genetic data of participants who consent will be shared with the Brazilian Ministry of Health, allowing for more in-depth analyses to be carried out in future.

One of the strengths of this project will be the construction of a robust database that will allow a better understanding of the genomic profile of patients with breast and prostate cancer in Brazil. Considering the miscegenation of the Brazilian population, the knowledge generated from these data will have implications for future studies of this specific population worldwide. Moreover, the identification of genetic variants in specific patient groups through somatic and germinal exome findings may allow individualization of tumor management. This knowledge should inform the development of public policies for better management and treatment of breast and prostate cancers.

## 4 Ethics and dissemination

The study protocol was submitted and approved by the research ethics committee of the coordinating institution, HMV (CAAE 55457122.3.1001.5330), in early 2022. All participating centers will also be required to submit the study protocol for consideration by their local ethics committees. This clinical trial has been registered in a public trial registry (clinicaltrial.gov NCT05306600 on April 1, 2022). Patients who meet the eligibility criteria will be invited to participate in the study. All those who freely and spontaneously agree to participate must sign an informed consent form previously approved by the local ethics committee.

This study was designed and will be implemented and reported in accordance with the *Guidelines for Good Pharmacoepidemiology Practices* of the International Society of Pharmacoepidemiology and the International Council for Harmonization Good Clinical Practice (ICH GCP) Guideline. All investigators involved will follow the recommendations of Brazilian National Health Council Resolution 466/12, as well as any other local and international rules applicable to the conduct of this study. Likewise, the confidentiality of patient data will be preserved by all investigators; after data collection, only information that does not allow patient identification at the individual level will be used. All results from this study will be published in international scientific journals.

## Ethics statement

The studies involving humans were approved by Hospital Moinhos de Vento (CAAE 55457122.3.1001.5330). The studies were conducted in accordance with the local legislation and institutional requirements. The participants provided their written informed consent to participate in this study.

## Author contributions

JS: Investigation, Methodology, Project administration, Writing – original draft. CB: Conceptualization, Investigation, Methodology, Writing – review & editing. MR: Conceptualization, Investigation, Methodology, Writing – review & editing. AD: Investigation, Methodology, Project administration, Writing – review & editing. MB: Investigation, Methodology, Project administration, Supervision, Writing – review & editing. GM: Conceptualization, Investigation, Methodology, Supervision, Writing – review & editing. DR: Conceptualization, Funding acquisition, Investigation, Methodology, Supervision, Writing – review & editing.
